# Identification and immuno-infiltration analysis of cuproptosis regulators in human spermatogenic dysfunction

**DOI:** 10.3389/fgene.2023.1115669

**Published:** 2023-03-29

**Authors:** Ming Zhao, Wen-Xiao Yu, Sheng-Jing Liu, Ying-Jun Deng, Zi-Wei Zhao, Jun Guo, Qing-He Gao

**Affiliations:** ^1^ Graduate School, Beijing University of Chinese Medicine, Beijing, China; ^2^ Department of Andrology, Xiyuan Hospital of China Academy of Chinese Medical Sciences, Beijing, China

**Keywords:** spermatogenic dysfunction, male infertility, cuproptosis, molecular clusters, immune infiltration

## Abstract

**Introduction:** Cuproptosis seems to promote the progression of diverse diseases. Hence, we explored the cuproptosis regulators in human spermatogenic dysfunction (SD), analyzed the condition of immune cell infiltration, and constructed a predictive model.

**Methods:** Two microarray datasets (GSE4797 and GSE45885) related to male infertility (MI) patients with SD were downloaded from the Gene Expression Omnibus (GEO) database. We utilized the GSE4797 dataset to obtain differentially expressed cuproptosis-related genes (deCRGs) between SD and normal controls. The correlation between deCRGs and immune cell infiltration status was analyzed. We also explored the molecular clusters of CRGs and the status of immune cell infiltration. Notably, weighted gene co-expression network analysis (WGCNA) was used to identify the cluster-specific differentially expressed genes (DEGs). Moreso, gene set variation analysis (GSVA) was performed to annotate the enriched genes. Subsequently, we selected an optimal machine-learning model from four models. Finally, nomograms, calibration curves, decision curve analysis (DCA), and the GSE45885 dataset were utilized to verify the predictions’ accuracy.

**Results:** Among SD and normal controls, we confirmed that there are deCRGs and activated immune responses. Through the GSE4797 dataset, we obtained 11 deCRGs. ATP7A, ATP7B, SLC31A1, FDX1, PDHA1, PDHB, GLS, CDKN2A, DBT, and GCSH were highly expressed in testicular tissues with SD, whereas LIAS was lowly expressed. Additionally, two clusters were identified in SD. Immune-infiltration analysis showed the existing heterogeneity of immunity at these two clusters. Cuproptosis-related molecular Cluster2 was marked by enhanced expressions of ATP7A, SLC31A1, PDHA1, PDHB, CDKN2A, DBT, and higher proportions of resting memory CD4^+^ T cells. Furthermore, an eXtreme Gradient Boosting (XGB) model based on 5-gene was built, which showed superior performance on the external validation dataset GSE45885 (AUC = 0.812). Therefore, the combined nomogram, calibration curve, and DCA results demonstrated the accuracy of predicting SD.

**Conclusion:** Our study preliminarily illustrates the relationship between SD and cuproptosis. Moreover, a bright predictive model was developed.

## Introduction

The World Health Organization (WHO) reported that infertility accounts for about 10%–15% of couples of child-bearing ages, with the male factor accounting for about 50% (Practice Committee of the American Society for Reproductive Medicine, 2015). In recent years, the prevalence of infertility among couples at child-bearing age in industrialized countries has reached 15%–20% (Choy and Eisenberg, 2018). Male infertility (MI) is described as the inability of the female partner to conceive naturally due to male factors after the couple has lived together for more than 12 months without using any contraception (Kunisaki et al., 2022). With the development of society, MI is on the rise year by year, which undoubtedly burdens individuals, families, society, and the globe (Sun et al., 2017). Notably, it is common knowledge that the spermatogenesis function of the testes is the direct cause of semen quality. Spermatogenic dysfunction (SD) may give rise to MI. The fact that the spermatogenic function of the testis is issued as part of complex genetic regulation has attracted extensive interest. Therefore, exploring associated regulators at the genomic level may be very important to treat MI precisely.

Nowadays, many genes associated with cell death have been identified that may play a surprising role in the process of testicular spermatogenesis. In mouse testicular germ cells, Bai et al. (2022) found that overexpression of FOXJ2 upregulates LAMP2A, which activates aberrant autophagy and leads to failure of spermatogenesis at the onset of meiosis, consequently contributing to MI. Gpx4, a crux upstream regulator of ferroptosis, attenuates its expression contributing to the restoring male fertility (Ingold et al., 2015). Moreover, NLRP3 inflammasome-mediated pyroptosis is regulated by a series of pyroptosis-related genes that may adversely affect male fertility (Perri et al., 2022). Thus, these collective findings suggest that cell death (CD) influence in testicular spermatogenesis cannot be ignored. However, whether some other new modes of CD and their associated molecular signatures play a role remains unclear and needs further exploration.

Copper is involved in human life activities as a trace element and cofactor for many essential enzymes, characterized by powerful redox activity and protein binding capacity. Functionally, copper is involved in regulating the physiological functions of cells *via* maintaining intracellular copper homeostasis. Stimulation by exogenous factors can trigger an imbalance in intracellular copper metabolism, mediating cytotoxicity and ultimately damaging the organism (Kim et al., 2008). However, copper homeostasis is associated with spermatogenesis (Herman et al., 2020). It is common knowledge that regulating the process of regulated cell death (RCD) is critical in determining cell fate (Nirmala and Lopus, 2020). Notably, cuproptosis is a novel RCD model that differs from the known RCDs (e.g., apoptosis, necroptosis, pyroptosis, ferroptosis, etc.,) and relies mainly on mitochondrial respiration. In mitochondrial respiration, copper ions (Cu^2+^) bind to lipid-acylated components of the tricarboxylic acid (TCA) cycle, leading to lipid-acylated protein aggregation and subsequent downregulation of Fe-S cluster proteins, which results in proteotoxic stress and finally CD (Tsvetkov et al., 2022). Additionally, FDX1 and proteolipid acylation are critical factors in regulating of cuproptosis. Cu^2+^ binding to the lipoylation-modified TCA cycle regulates their oligomerization and causes Fe-S cluster protein deficiency, mediating cuproptosis. Moreover, it has been observed that cuproptosis-induced damage can be attenuated by inhibiting mitochondrial pyruvate carriers and electron transport chain activity (Cobine and Brady, 2022; Tang et al., 2022; Tsvetkov et al., 2022). Furthermore, many recent studies suggest that abnormal energy metabolism and oxidative stress induced by mitochondrial dysfunction may be decisive factors involved in SD (Cheng et al., 2018; Aitken and Drevet, 2020; Baskaran et al., 2021).

The pathogenesis of SD, however, remains unclear. Therefore, we hypothesized that cuproptosis-related genes (CRGs) are inextricably linked to the development of SD. In our study, we explored our hypothesis through a bioinformatics approach to contribute to understanding SD.

## Materials and methods

### Data source and pre-processing

To obtain SD-related RNA-seq and clinical data, we searched and screened the Gene Expression Omnibus (GEO) database (https://www.ncbi.nlm.nih.gov/geo) as of 10 November 2022, using the search term “male infertility.” Species were set to “*Homo sapiens*”, by selecting “series”, a total of 65 series were screened out. Inclusion criteria: 1) mRNA expression profiles by array or high-throughput sequencing; 2) tissue samples were derived from human testicular tissue; 3) normal control and SD patient groups were included in the dataset; 4) spermatogenic blockage occurred at either stage in the SD patient group. Exclusion criteria: 1) duplicate datasets; 2) datasets lacking SD sample groups; 3) datasets lacking normal control sample groups; 4) non-testicular tissue samples; 5) studies or data on animals or cell lines. Based on this, we retrieved a total of two eligible datasets. The two microarray datasets (GSE4797 and GSE45885) related to SD were downloaded from the GEO database. The GSE4797 dataset (GPL2891 Platform) including 12 healthy (full spermatogenesis) and 16 SD (spermatid stage arrest, spermatocyte stage arrest, and Sertoli-cell-only syndrome) testicular tissue samples were selected for analysis. The GSE45885 dataset (GPL6244 Platform), which included testicular tissue from 4 normal (full spermatogenesis) subjects and 27 SD (postmeiotic arrest, meiotic arrest, and Sertoli-cell-only syndrome) samples, were selected for validation. The raw data of gene expression matrices from GEO datasets were processed and normalized *via* the R package of “limma” (version 3.52.4).

### Evaluation of immune cell infiltration

Using the CIBERSORT algorithm (https:/cibersort.stanford.edu/) and LM22 signature matrix, the relative abundance of 22 types of immune cells in each sample was calculated according to available gene expression data. CIBERSORT was used to obtain the inverse convolution *p*-value for each sample using Monte Carlo sampling. The transcriptional feature matrix of the 22 immune cells was also used to simulate the calculation. The sum of the percentages of 22 immune cells in each sample was 1. Here, we set the number of simulations calculated to 1,000, and samples with a *p*-value <0.05 were identified as the exact immune cell subtype.

### Correlation analysis of CRGs and immune cell infiltration

Spearman correlation analysis was used to determine the correlation coefficient between CRGs and the relative abundance of immune cells to illustrate the relationship between CRGs and SD-related immune cell characteristics. *p*-values less than 0.05 were considered to be significantly correlated. The result is presented *via* the R package of “ggplot2” (version 3.4.0).

### Cluster analysis of SD patients

The optimal number of clusters was evaluated based on a combination of cumulative distribution function (CDF) curve, consistent clustering score, and consensus matrix. We set the maximum number of fractals k = 9.

### Gene set variation analysis (GSVA)

GSVA enrichment analysis was performed using the R package of “GSVA” (version 1.44.5). We obtained the files “c2. cp.kegg.symbols” and “c5. go.symbols” from the MSigDB website database (http://www.gsea-msigdb.org/gsea/msigdb). GSVA scores were calculated using the R package “limma” (version 3.52.4), and |t value of GSVA scores| > 2 were considered to be significantly altered.

### Weighted gene co-expression network analysis (WGCNA)

WGCNA used the R package of “WGCNA” (version 1.71) to identify co-expression modules. The top 25% of genes with the most considerable fluctuations were selected for subsequent WGCNA analysis to ensure the accuracy of the results. Firstly, the Pearson correlation coefficient between two genes was calculated, and the gene expression similarity matrix was constructed accordingly. Secondly, the gene expression similarity matrix is transformed into an adjacency matrix, and a soft threshold power *β* generates an enhanced adjacency matrix. Thirdly, the Pearson correlation analysis constructed an unsupervised gene co-expression relationship matrix with an enhanced adjacency matrix. Based on the topological overlap measure (TOM), an indicator of the degree of association between genes, it was turned into a topological matrix. Fourthly, based on the inter-gene dissimilarity (1-TOM), genes with similar gene expression patterns were divided into the same gene module using the average linkage hierarchical clustering method. Additionally, a dynamic shearing algorithm was used to determine gene co-modules from the systematic clustering tree, and gene modules with similarity higher than 75% were merged. Finally, the relationship between module membership (MM) and gene significance (GS) was computed for each gene within the target module to verify the reliability of the selected module.

### Construct predictive models based on multiple machine-learning methods

We used the R package of “caret” (version 6.0.93) to build machine-learning models based on two different clusters of CRGs, which include support vector machine (SVM), eXtreme Gradient Boosting (XGB), generalized linear model (GLM) and random forest model (RF). SVM is a class of generalized linear classifiers that perform binary data classification through supervised learning. It is unique in solving small samples, non-linear and high-dimensional pattern recognition (Krause et al., 2007). XGB can be understood as a parallel prediction model with multiple trees, which iterates continuously, generating a new tree at each iteration, so that the predicted values keep approximating the true values (Babajide Mustapha and Saeed, 2016). GLM, on the other hand, establishes the mathematical expectations of the response variables through link functions and can predict the relationship between linear combinations of variables (Takada et al., 2017). Furthermore, RF is an integrated machine-learning approach that uses a variety of autonomous decision trees to predict classification or regression (Rigatti, 2017).

Distinct clusters are used as response variables, and cluster-specific DEGs are selected as explanatory variables. The 16 SD samples were randomly divided into a training set (N = 11) and a validation set (N = 5) in a ratio of 7 to 3. The R package of “caret” automatically adjusted the parameters of these machine learning models *via* grid search, where we all perform using default parameters. Subsequently, evaluation was performed by 5-fold cross-validation. The R package “DALEX” (version 2.4.2) was then used to interpret the four machine learning models and visualize the residual distributions and feature importance. The R package of “ pROC” (version 1.18.0) was used to visualize the area under the curve (AUC) of the receiver operating characteristic (ROC). After identifying the best machine learning model, the first 5 key variables were the predictor genes that can be used as SD correlations. Finally, the ROC curve results of the GSE45885 dataset were used to validate the diagnostic value of the machine learning model.

### Construction of the nomogram model and the independent validation analysis

The R package “rms” (version 6.3.0) was used to construct the nomogram model, with each predictor having a corresponding score and the “total score” being the sum of the scores of the predictors. Additionally, the predictive power of the nomogram model was estimated using calibration curves and decision curve analysis (DCA). The external dataset GSE45885 was used to validate the ability of the prediction model to distinguish between SD and normal controls. Furthermore, the ROC curves were visualized using the R package “pROC”.

### Statistical analysis

Continuous variables are presented as mean ± standard deviation, while categorical variables are expressed as percentages. R software (Version 4.2.0, https://www.r-project.org/) was used for all data analysis and statistical analyses in this study. The Wilcox test was used to assess whether there was a statistical difference between the two groups. The relationship between CRGs expression levels and immune cells was analyzed using the Spearman correlation analyses. We considered the result statistically significant when the *p*-value was less than 0.05.

## Results

### CRGs expression and immune infiltration in SD patients

**FIGURE 1:**
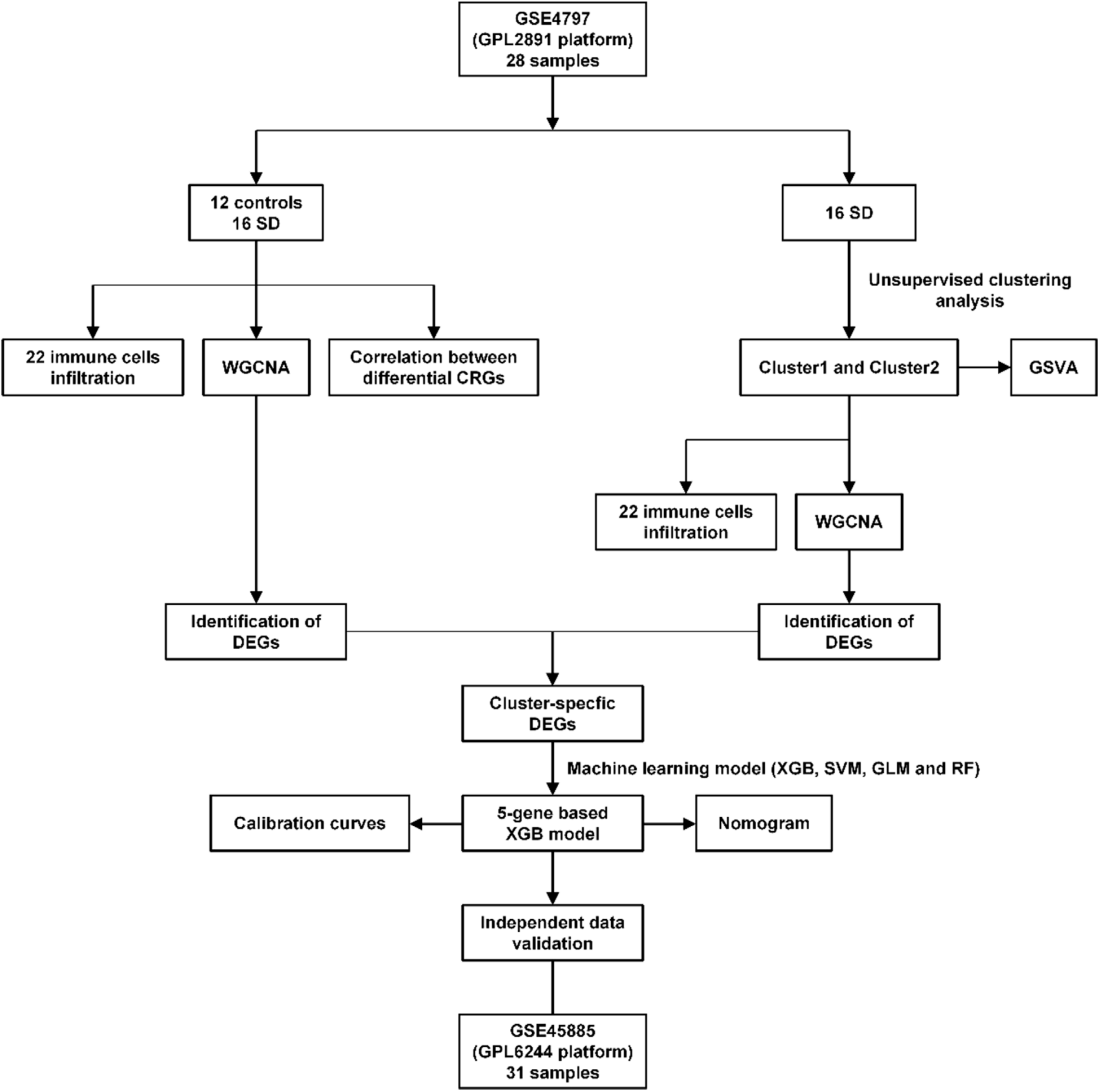
Flow chart of this study.

**FIGURE 2:**
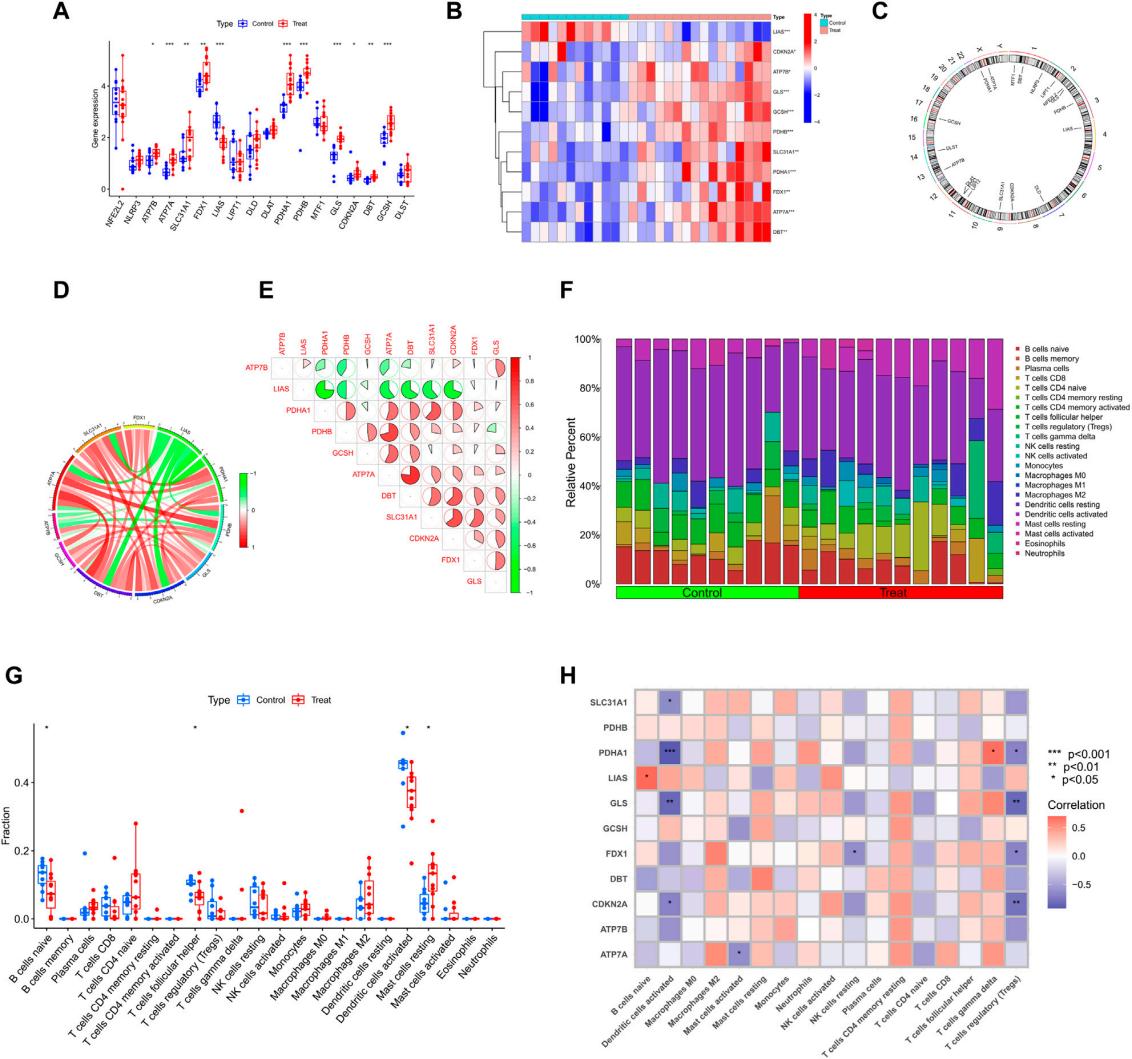
Identification of deCRGs in SD **(A)** The expression levels of 18 CRGs among spermatogenic normal and dysfunctional testicular tissues in the GEO database **(B)** Heatmap of deCRGs **(C)** The location of 18 CRGs on chromosomes **(D)** Gene relationship network diagram of 11 deCRGs **(E)** Correlation analysis of 11 deCRGs. Red and green colors represent positive and negative correlations, respectively. The correlation coefficient was expressed as the area of the pie chart **(F)** The relative abundance of 22 infiltrated immune cells between normal and dysfunctional testicular tissues **(G)** Boxplots of differences in immune infiltration between SD and normal controls **(H)** Heatmap showed the correlation between 11 deCRGs and infiltrated immune cells. *p < 0.05, **p < 0.01, ***p < 0.001.

To explore the correlation between cuproptosis and testicular spermatogenic function, we retrieved a total of 65 series. Based on the inclusion and exclusion criteria, only two datasets met the requirements. Therefore, patients with SD from the GEO cohort (GSE4797 and GSE45885) were enrolled (Supplementary Table S1). Figure 1 illustrates the flow of our study. We identified 11 differentially expressed CRGs (deCRGs). Among them, the expression levels of ATP7A, ATP7B, SLC31A1, FDX1, PDHA1, PDHB, GLS, CDKN2A, DBT, and GCSH were higher. In contrast, the expression level of LIAS was largely lower in the testicular tissues of SD than in normal controls (Figures 2A, B). Figure 2C shows the locations of CRGs on chromosomes. To investigate the role of CRGs in the progression of SD, we performed a correlation analysis of these deCRGs. Notably, the gene relationship network diagram showed a close relationship between these deCRGs (Figure 2D). Among them, ATP7A and DBT showed a strong synergistic effect (coefficient = 0.77), while PDHA1 and LIAS showed a significant antagonistic effect (coefficient = −0.74). Furthermore, most of these genes showed a positive correlation with each other (Figure 2E).

Figure 2F shows the immune infiltration analysis based on the CIBERSORT algorithm, demonstrating the difference in the proportion of 22 infiltrated immune cell types between the two groups, and therefore was employed to elucidate whether there are differences in the immune system between SD and normal controls. Figure 2G shows that SD patients presented higher levels of mast cell resting infiltration and normal controls had higher levels of infiltration of the B-cell naive phase, T-cell follicular helper phase, and dendritic cell activation. Meanwhile, the correlation between immune cell infiltration and deCRGs is represented in Figure 2H.

### Identification of cuproptosis clusters in SD

**FIGURE 3:**
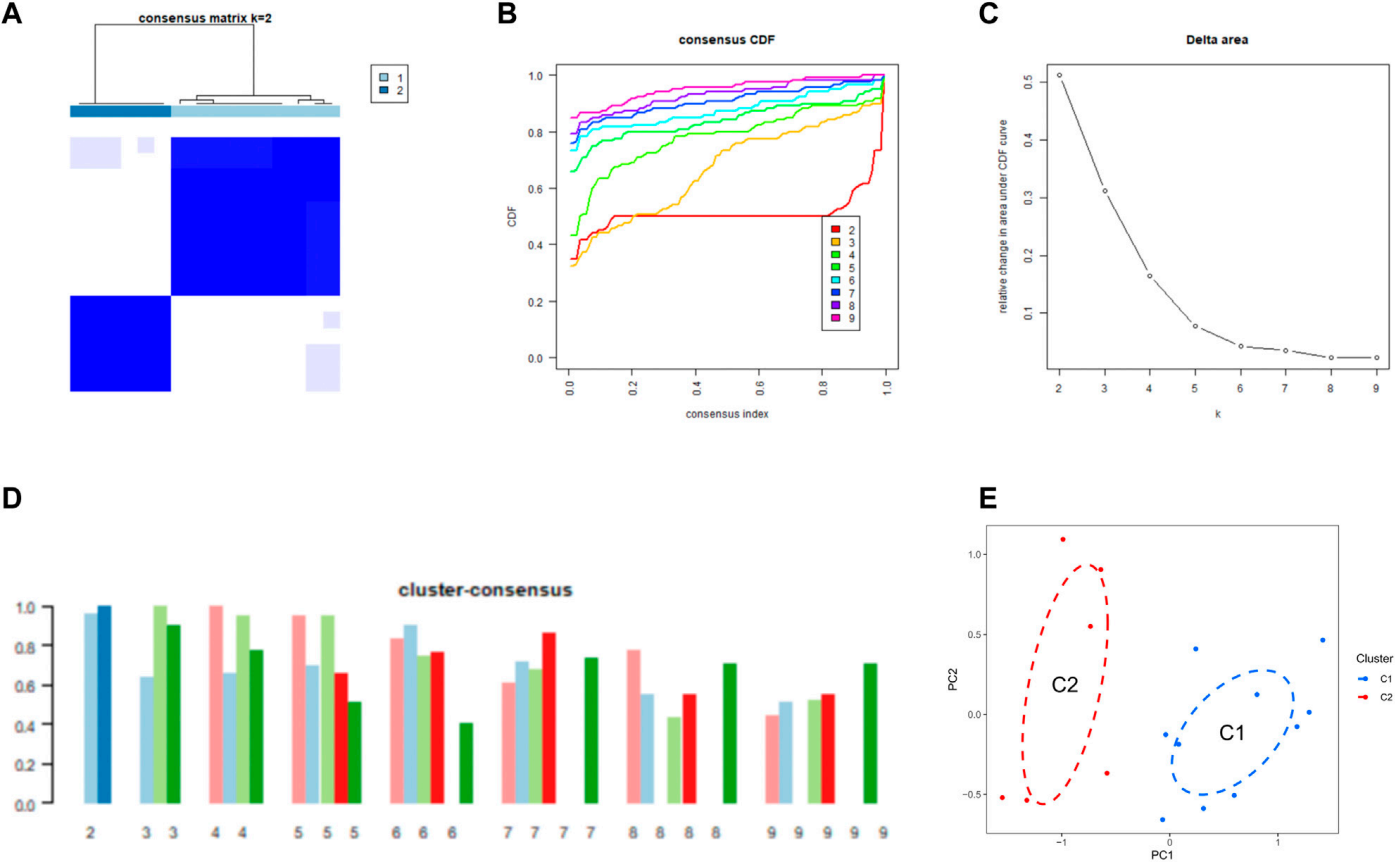
Identification of CRG clusters in SD **(A)** Consensus clustering matrix when k = 2 **(B–D)** Representative CDF curves **(B)**, CDF delta area curves **(C)**, and the score of cluster-consensus **(D) (E)** PCA visualized the distribution of the two clusters.

We used a consensus clustering algorithm to group 16 SD samples based on the expression profiles of 11 CRGs, by which we elucidated the expression patterns associated with cuproptosis in SD. The number of clusters was most stable when k = 2, and the CDF curve fluctuated between 0.2 and 0.6 (Figures 3A, B). Figure 3C shows the area under the CDF curve when k = 2 to 9. When k = 2, the consistency score of each subtype was the largest and closest (Figure 3D). Figure 3E shows a significant difference in the principal component analysis (PCA) between these two clusters.

### Differences in CRGs and immune infiltration characteristics between two clusters

**FIGURE 4:**
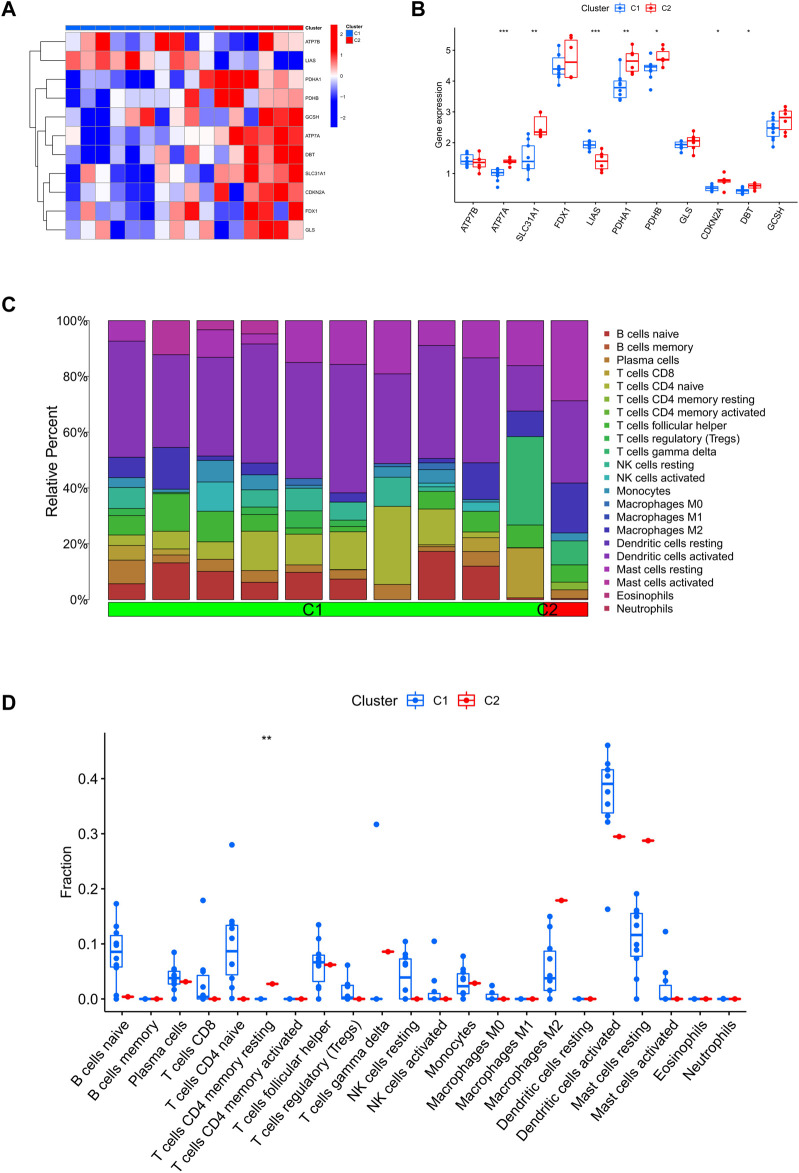
Identify the molecular and immunological features between the two clusters. **(A)** Heatmap of the expression patterns of the 11 CRGs between the two clusters. **(B)** Boxplots of 11 CRGs expressed between the two clusters. **(C)** Relative abundance maps of 22 infiltrating immune cells between the two clusters. **(D)** Boxplots of immune infiltration differences between the two clusters.

Firstly, we assessed the expression differences of 11 CRGs between Cluster1 and Cluster2 to explore the molecular features between clusters. We observed a different CRG expression profile between the two cuproptosis clusters (Figure 4A). Cuproptosis Cluster1 showed high expression levels of LIAS, while Cuproptosis Cluster2 showed high levels of ATP7A, SLC31A1, PDHA1, PDHB, CDKN2A, and DBT (Figure 4B). In addition, Figure 4C shows that the immune microenvironment between cuproptosis Cluster1 and Cluster2 was altered. Furthermore, cuproptosis Cluster2 exhibited a higher proportion of CD4^+^ T cell memory rest (Figure 4D).

### Screening of gene modules and construction of co-expression networks

**FIGURE 5:**
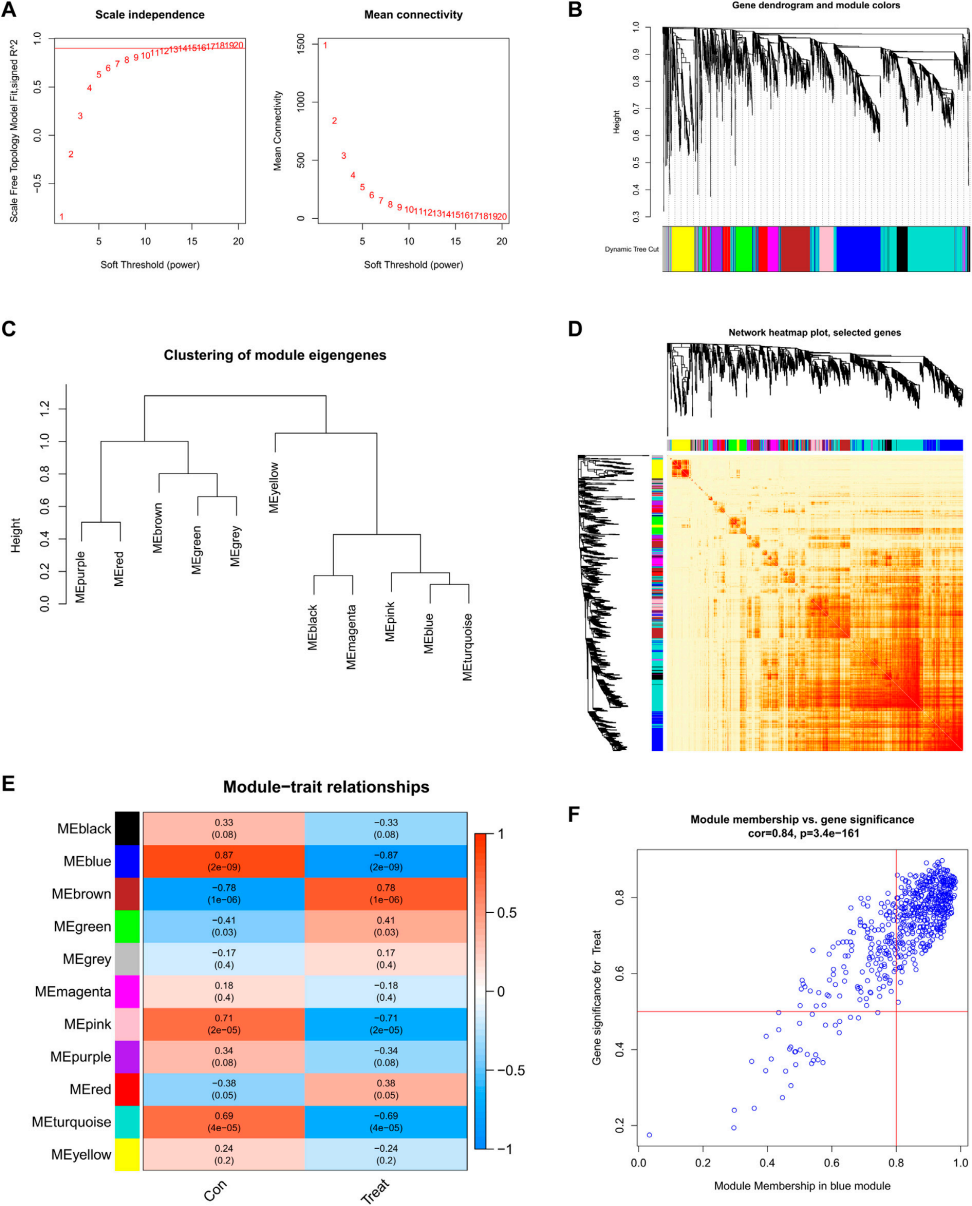
Co-expression network of DEGs in SD **(A)** Set soft threshold power **(B)** The cluster tree dendrogram of co-expression modules is shown in different colors **(C)** Cluster diagram of module eigengenes **(D)** TOM heatmap of 11 modules **(E)** Heatmap of correlation analysis of module eigengenes with clinical features. Rows and columns represent modules and clinical features, respectively **(F)** Scatter plot of the genetic significance of the blue module members with SD.

We established a co-expression network of normal controls and SD patients by WGCNA and identified key gene modules associated with SD. When the soft threshold power was 12, the scale-free R^2^ approached 0.9, and co-expressed gene modules under this condition were identified (Figure 5A). Subsequently, the dynamic cutting algorithm obtained a total of 11 co-expression modules with different colors and a TOM heatmap (Figures 5B-D). In addition, similarities and contiguities in the co-expression of module-clinical features (normal control and SD) were analyzed by applying the genes in these 11 modules. We found the strongest association of the blue module with SD, which includes 431 genes (Figure 5E). Moreso, there was a positive correlation between the blue module and the module-related genes (Figure 5F).

**FIGURE 6:**
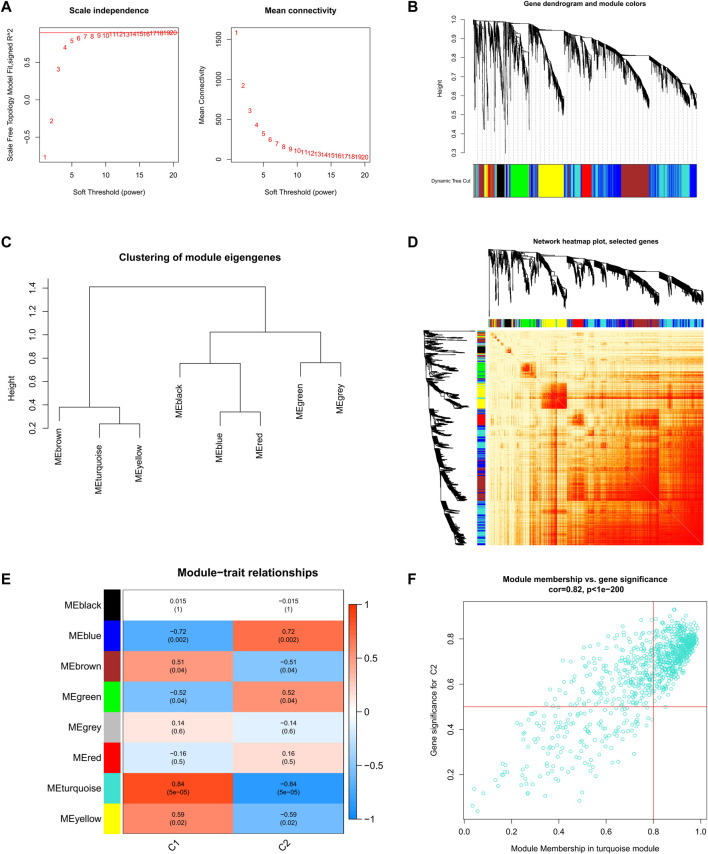
Co-expression network of DEGs between the two cuproptosis clusters **(A)** Set soft threshold power **(B)** The cluster tree dendrogram of co-expression modules is shown in different colors **(C)** Cluster diagram of module eigengenes **(D)** TOM heatmap of 8 modules **(E)** Heatmap of correlation analysis of module eigengenes with clinical features. Rows and columns represent modules and clinical features, respectively **(F)** Scatter plot of the genetic significance of the turquoise module members with Cluster1.

Further, we analyzed key gene modules closely related to the cuproptosis clusters by WGCNA. When the soft threshold parameter *β* = 9, R^2^ = 0.9, and the scale-free network was constructed under this condition (Figure 6A). Eight modules were identified as important and contained 3624 genes, and the heatmap shows the TOM of all module-related genes (Figures 6B-D). Analysis of the relationship between modules and clinical traits (Cluster1 and Cluster2) revealed a high correlation between turquoise modules (545 genes) and SD clusters (Figure 6E). Furthermore, the results of the correlation analysis showed a significant positive correlation between the turquoise modules and the corresponding genes (Figure 6F).

### Identification and functional annotation of cluster-specific DEGs

**FIGURE 7:**
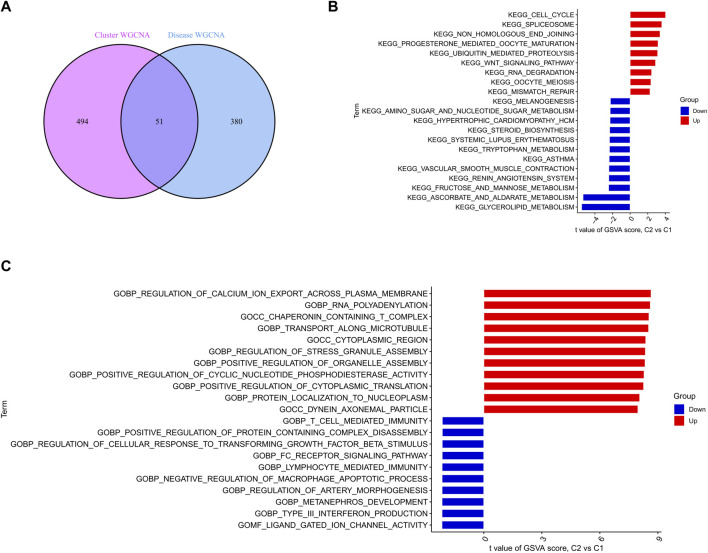
Identification of specific clusters of DEGs and their biological functions **(A)** Crossover genes of the cuproptosis clusters module and the SD module **(B–C)** Differences in hallmark pathway **(B)** and biological functions **(C)** activities between upregulated and downregulated DEGs ranked by t-value of GSVA.

Five hundred forty-five module-related genes of the cuproptosis clusters and 431 module-related genes of SD were intersected to obtain a total of 51 cluster-specific DEGs (Figure 7A). Enrichment of these genes for pathways and biological functions was performed by GSVA analysis. The enrichment results of the pathways revealed that cell cycle, spliceosome, and non-homologous end joining were reinforced in upregulated DEGs. At the same time, glycerolipid metabolism, and ascorbate and aldarate metabolism activity were enhanced in downregulated DEGs (Figure 7B). Notably, the functional enrichment esults showed that the upregulated genes were remarkably related to regulating calcium ion export across the plasma membrane, RNA polyadenylation, and transport along the microtubule (Figure 7C).

### Building machine learning models

**FIGURE 8:**
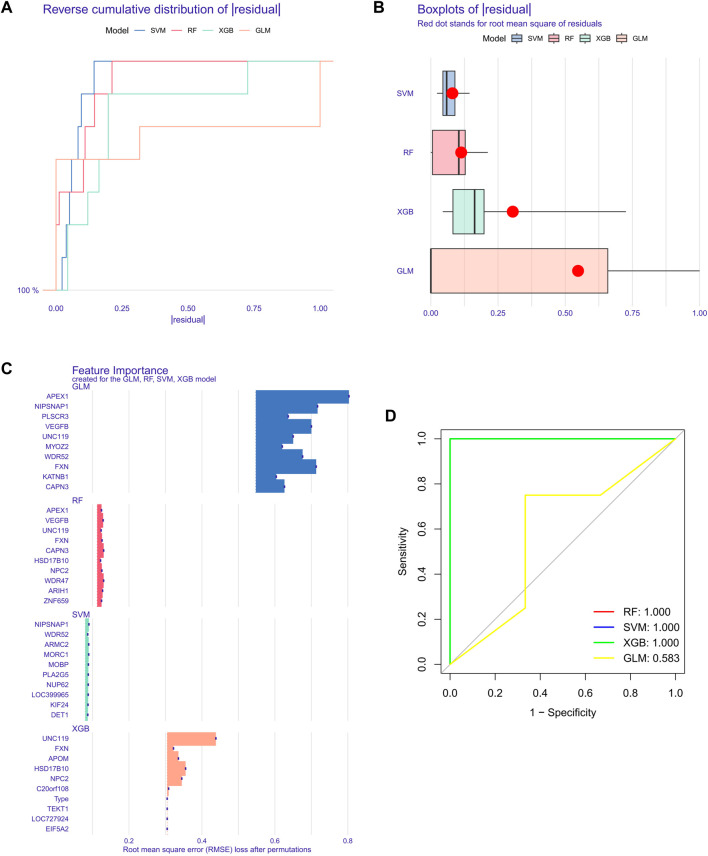
Construction of SVM, RF, XGB, and GLM machine models. **(A)** The cumulative residual distribution of the four models. **(B)** Residual Boxplots of the four machine learning models, where the red dots indicate the root mean square of the residuals **(C)** The important features in SVM, RF, XGB, and GLM. **(D)** ROC analysis of four machine learning models with 5-fold cross-validation in the test set.

To further screen out specific genes with high diagnostic value, we built four machine learning models *via* the R package of “caret”, SVM, XGB, GLM, and RF, based on the expression profiles of 51 cluster-specific DEGs in the SD training set. The results of the residual distribution of the four models show that GLM had the highest residuals, while SVM, RF, and XGB had relatively low residuals (Figures 8A, B). Figure 8C ranks each model’s top 15 significant characteristic variables according to the root mean square error (Figure 8C). To further evaluate the discriminative performance of the four machine learning algorithms in the test set, we computer-plotted the ROC curves for 5-fold cross-validation. RF, SVM, and XGB machine learning models, have an AUC of 1, while GLM’s was only 0.583 (Figure 8D). In summary, XGB was selected as the best model because we believe it can best distinguish between different patient populations. Finally, we identified five genes, FXN, APOM, NPC2, HSD17B10, and UNC119, in the XGB model as the most critical predictive genes for further analysis.

### Evaluating machine learning models

**FIGURE 9:**
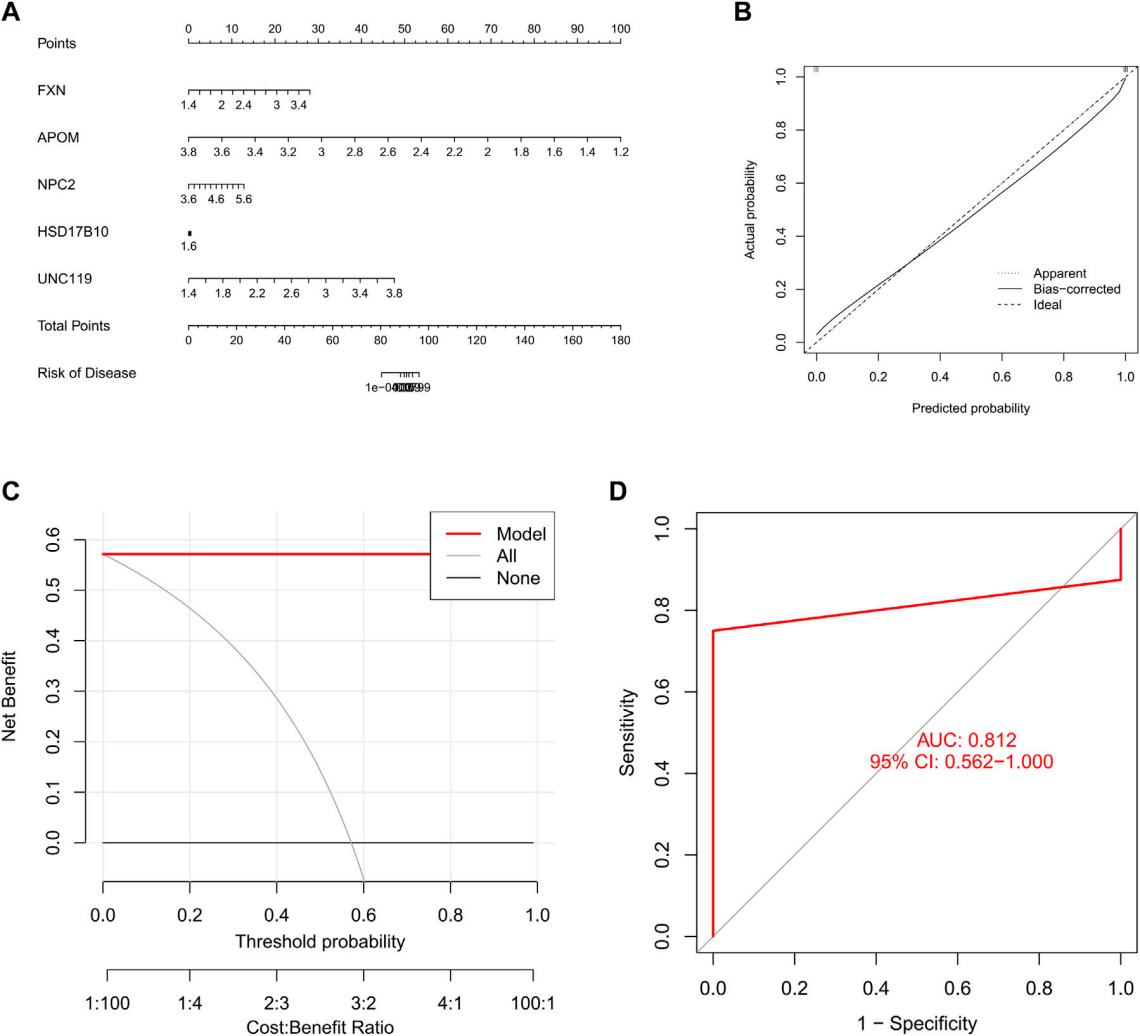
Validation of a 5-gene-based XGB model. **(A)** Construction of a nomogram to predict SD risk based on a 5-gene XGB model **(B, C)** Calibration curves **(B)** and DAC **(C)** to assess the predictive ability of the nomogram model **(D)** ROC of the 5-gene-based XGB model in GSE45885 datasets.

We constructed a nomogram to assess the risk of cuproptosis clusters in 16 SD patients, and its primary purpose was to evaluate the predictive performance of the XGB model (Figure 9A). The prediction efficiency of the nomogram model was assessed using calibration curves and DCA. The calibration curves show minimal error between the actual SD clustering risk and the predicted risk (Figure 9B). Additionally, the DCA demonstrated that the nomogram has high accuracy and can provide a reference for clinical decision-making (Figure 9C). Subsequently, we validated the prediction model for 5-gene on another dataset including testicular tissue from normal controls and SD patients. The AUC of 0.812 for the GSE45885 dataset illustrates the satisfactory performance of the 5-gene prediction model (Figure 9D), implying that our diagnostic model helps distinguish SD from normal controls.

## Discussion

MI is an illness caused by multiple factors leading to male reproductive dysfunction, and the SD of the testes is one of the critical causes (Skakkebaek et al., 1994; Krausz, 2011). The typical phenotype of SD severely impaires spermatogenesis, leading to azoospermia or severe oligospermia, with genetic factors often being the underlying cause of this phenomenon (Song et al., 2016). Studying the CD process in male germ cells at the genetic level will reveal the relationship between the interactions of various factors. The use of individualized treatment modalities in reproductive medicine also provides opportunities for the treatment of MI. Therefore, the role of finding a more appropriate molecular cluster to guide the development of a personalized treatment plan for SD-induced MI cannot be underestimated (Arato et al., 2020). Cuproptosis is a recently reported copper-dependent mode of CD, mainly manifested by the aggregation of lipidated mitochondrial enzymes, and is closely associated with disease progression (Tsvetkov et al., 2022). However, the precise mechanisms of regulation of SD by cuproptosis have not been uncovered. Therefore, we in this study attempted to elucidate the role of CRGs in SD and immune microenvironments utilizing bioinformatics analysis. Furthermore, we also used the gene signature associated with cuproptosis to predict SD subtypes.

Firstly, we analyzed the expression of CRGs in the testicular tissue of SD patients and normal controls. Expression levels of 11 CRGs were significantly up-or downregulated in SD, including ATP7A, ATP7B, SLC31A1, FDX1, PDHA1, PDHB, GLS, CDKN2A, DBT, GCSH, and LIAS, indicating that CRGs play a significant role in the development of SD. Moreso, CDKN2A is associated with cellular processes in germ cells under the regulation of long non-coding RNA (lncRNA) (Joshi and Rajender, 2020).

Secondly, we calculated the correlation between these deCRGs to further explore the crosstalk between cuproptosis and SD. Our research also found significant synergistic or antagonistic effects between some cuproptosis regulators. In addition, the relative abundance of immune cells was changed between SD patients and normal controls. SD patients presented higher infiltration levels of mast cells resting. Normal controls had a higher infiltration level of B cells naive, T cells follicular helper, and dendritic cells activated. This suggests to us that alterations in the function of some immune cells in the testicular immune microenvironment may play an important role in spermatogenesis. Using unsupervised cluster analysis, the expression landscape based on CRGs was further used to illustrate the different modes of cuproptosis regulation in SD, resulting in the identification of two different CRG clusters. Cluster2 is characterized by a higher immune fraction and a relatively high level of immune infiltration, indicating that its immune microenvironment is differs from Cluster1. Additionally, Cluster2 was characterized by enhanced expressions of ATP7A, SLC31A1, PDHA1, PDHB, CDKN2A, and DBT, and higher proportions of resting memory CD4^+^ T cells.

In the experimental autoimmune orchitis model characterized by testicular spermatogenic dysfunction, Nagahori et al. observed numerous CD4^+^ T cells around the seminiferous tubules (Nagahori et al., 2021). In Cluster2, overexpression of CRGs further illustrates the relevance of cuproptosis to immune infiltration. SLC31A1 is mainly localized in the plasma membrane, and its role is to regulate a certain range of intracellular Cu^2+^ concentration, which is associated with the development of immune function (Yu et al., 2019). Lu et al. found that SLC31A1 can regulate the cell viability, proliferation, cycle progression, and activation of CD4^+^ T cells (Lu et al., 2021). Overexpression of PDHA1 increases reactive oxygen species (ROS) production, mitochondrial respiration, and apoptosis (Wang et al., 2022), which may be a driver of the disturbance of the testicular immune microenvironment. Increased expression of CDKN2A correlates with a lymphocyte phenotype associated with immunosenescence (Bourlon et al., 2020). Although the mechanisms of CRGs in SD immune regulation are less studied, it can be speculated based on previous studies and the results of this study that cuproptosis may play an important role in SD immune infiltration. How to prevent and treat SD by regulating the balance of copper in immune cells needs further in-depth study. In addition, fifty-one cluster-specific DEGs indicated that cell cycle, spliceosome, and non-homologous end joining were reinforced in upregulated DEGs. At the same time, glycerolipid metabolism, and ascorbate and aldarate metabolism activity were enhanced in downregulated DEGs. In conjunction, we speculated that Cluster2 might be more closely related to the progression of SD.

As research has progressed and evolved, machine learning models have been increasingly used to predict sperm parameters in MI (Zeadna et al., 2020; Ory et al., 2022). Compared with traditional univariate analysis, machine learning usually uses multi-factor analysis methods, which fully consider the relationships between variables. Therefore, machine learning models are more accurate, and the results are more credible. The R package of “caret” we used is a comprehensive machine learning toolkit designed to solve prediction problems. The feature of this package is the ability to quickly get all the materials ready, including the whole process of data pre-processing, model training, and model prediction (Lee and Kim, 2022). We compared the prediction performance of four machine learning models, XGB, SVM, GLM, and RF. An XGB-based prediction model was constructed with very high predictive validity (AUC = 1) in the test set. Subsequently, we selected five important variables (FXN, APOM, NPC2, HSD17B10, and UNC119) to construct a 5-gene-based XGB model. FXN gene encodes frataxin, which is a mitochondrial protein, and with its decrease, mitochondrial metabolism is seriously disrupted (O'Connell et al., 2022). Importantly, mitochondria are closely related to the spermatogenic function of testis (Okuda et al., 2011), suggesting that FXN may be a potential therapeutic strategy for MI patients. APOM is a high-density lipoprotein-associated apolipoprotein, which is involved in the reverse transport of cholesterol (Mosialou et al., 2010). APOM expression is regulated by its gene. At present, many studies have shown that the APOM gene is related to diabetes, dyslipidemia, obesity, and other diseases (Zhang et al., 2017; Sramkova et al., 2019; Liu et al., 2020), which are high-risk factors leading to MI. Moreso, the NPC2 gene encodes NPC2, a small soluble protein that can transfer cholesterol at a terrific speed. NPC2 directly binds to free cholesterol in the internal lysosomal membrane to regulate the homeostasis of intracellular cholesterol (Abdul-Hammed et al., 2010). Notably, testis cholesterol efficiently protects the blood-testis barrier (BTB). The level of NPC2 protein in male mice exposed to low dose rate chronic radiation was significantly reduced, having potential BTB damage and immune infertility (Son et al., 2015). Moreover, the effects of HSD17B10 and UNC119 in testicular spermatogenesis and on MI have not been reported and deserve further study.

In an externally validated dataset, the 5-gene XGB-based model (AUC = 0.812) accurately predicted SD, demonstrating its good diagnostic value for SD. In addition, we constructed a nomogram model for diagnosing SD using five genes, FXN, APOM, NPC2, HSD17B10 and UNC119. Our results show the good predictive performance of the model, indicating that this model has potential for clinical application. Together, the 5-gene-based XGB model used to differentiate subtypes of SD is satisfactory.

Our study still has some limitations that need to be addressed in future research. First, our current study was performed based on a comprehensive bioinformatics analysis, although we applied an external dataset for validation, and additional clinical or experimental evaluation is required to make sure the different genes in human SD were involved in cuproptosis. Furthermore, a greater number of SD samples are needed to elucidate the accuracy of cuproptosis-related clusters. In addition, the potential relevance of CRGs and immune cell infiltration in SD needs to be further explored. Moreover, more detailed clinical characteristics are required to determine the performance of the predictive model.

## Conclusion

In summary, our study found a significant correlation between CRG and SD and between CRG and immune cells infiltrated in SD. In addition, some immune heterogeneity between the two clusters in SD was identified. Thus, machine learning models can accurately assess the subtypes of SD, and a 5-gene XGB-based model may be the best choice. Notably, our study illustrates a novel relationship between cuproptosis. Furthermore, it establishes a promising predictive model to assess the risk of cuproptosis subtypes.

## Data Availability

The original contributions presented in the study are included in the article/Supplementary Material, further inquiries can be directed to the corresponding authors.
