# Supplementary material for: Identification and immuno-infiltration analysis of cuproptosis regulators in human spermatogenic dysfunction
Source: Front Genet. 2023 Mar 29;14:1115669. doi: 10.3389/fgene.2023.1115669 (PMC10090386; doi:10.3389/fgene.2023.1115669)
Supplement: Supplementary file 1 [file Table1.docx]

**Supplementary Table S1 Clinical and demographic characteristics of the patients from the GEO cohort**

|  | All subjects | SD | Controls |
| --- | --- | --- | --- |
| **GSE4797** |  |  |  |
| Patients | 28 | 16 | 12 |
| Testis region |  |  |  |
| *Right* | - | - | - |
| *Left* | - | - | - |
| *N/A* | 28 | 16 | 12 |
| *Pull-right and left* | - | - | - |
| Histopatological description |  |  |  |
| *Full spermatogenesis* | 12 | 0 | 12 |
| *Spermatid stage arrest*  *spermatocyte stage arrest*  *Sertoil Cell Only-Syndrome* | 6  5  5 | 6  5  5 | 0  0  0 |
| Age(years) |  | 32.6 ± 7.0 | 39.1 ± 7.0 |
| **GSE45885** |  |  |  |
| Patients | 31 | 27 | 4 |
| Testis region |  |  |  |
| *Right* | 15 | 15 | 0 |
| *Left* | 11 | 11 | 0 |
| *N/A* | 4 | 0 | 4 |
| *Pull-right and left* | 1 | 1 | 1 |
| Histopatological description |  |  |  |
| *Full spermatogenesis* | 4 | 0 | 4 |
| *Meiotic arrest*  *Postmeiotic arrest*  *Sertoil Cell Only-Syndrome* | 7  13  7 | 7  13  7 | 0  0  0 |
| Age(years) |  | 41.0 ± 13.0 | 39.0 ± 25.0 |
